# Heterogeneous flow inside threads of low viscosity fluids leads to anomalous long filament lifetimes

**DOI:** 10.1038/s41598-019-43590-z

**Published:** 2019-05-08

**Authors:** Steffen M. Recktenwald, Simon J. Haward, Amy Q. Shen, Norbert Willenbacher

**Affiliations:** 1 Karlsruhe Institute of Technology, Institute for Mechanical Process Engineering and Mechanics, Gotthard-Franz-Straße 3, 76131 Karlsruhe, Germany; 2 Okinawa Institute of Science and Technology Graduate University, Micro/Nano/Biofluidics Unit, 1919-1 Tancha, Onna-son, Okinawa, 904-0495 Japan

**Keywords:** Fluid dynamics, Chemical engineering, Self-assembly, Rheology, Fluids

## Abstract

Formation and breakup of fluid threads is pervasive in nature and technology, where high extensibility of fluid filaments and extended filament lifetimes are commonly observed as a consequence of fluid viscoelasticity. In contrast, threads of low viscous Newtonian fluids like water rupture quickly. Here, we demonstrate that a unique banding instability during filament thinning of model surfactant solutions, with a viscosity close to water and no measurable elasticity, leads to extremely long filament lifetimes and to the formation of remarkably long threads. Complementary measurements in planar extension as well as in shear reveal that this flow instability is characterized by a multivalued stress, arising beyond a critical strain rate, irrespective of flow kinematics. Our work reports the first observation of such phenomena during extensional deformation and provides a unifying view on instabilities in complex flow fields.

## Introduction

When you put a drop of saliva between your thumb and index finger, then slowly pull your fingers apart, a slender fluid thread forms. This filament starts to thin, eventually forming a periodic pattern of small beads connected by small threads, before it finally breaks. This simple experiment shows how complex fluids like saliva, which contains naturally occurring polymers that cause viscoelasticity, respond to stretching or elongational deformation. The breakup of fluid threads is a dynamical process, omnipresent in nature and highly relevant for many technological operations such as atomization and spraying^1^, electrospinning^2^, or roll-coating^3^.

**Figure 1:**
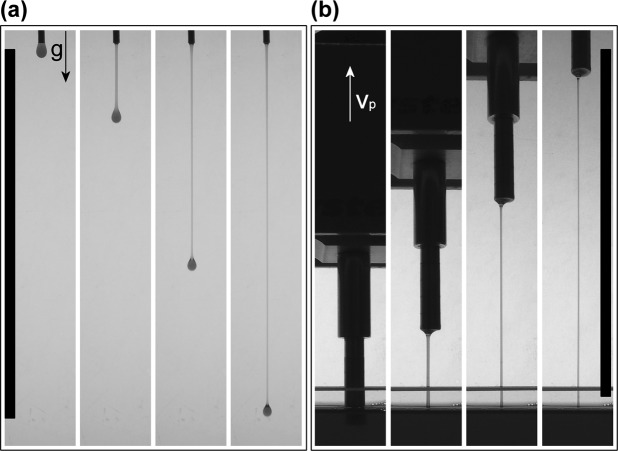
Formation of remarkably long surfactant filaments. Series of snapshots showing the formation of long filaments using a dilute surfactant solution (10 mM hexadecyltrimethylammonium bromide/sodium salicylate (CTAB/NaSal), R = 0.5). (**a**) A drop detaches due to gravity *g* from a blunt needle with diameter *D*_*n*_ = 1.65 mm, forming a long filament. (**b**) Cylindrical piston with diameter *D*_*p*_ = 6 mm moves upwards with a constant velocity *v*_*p*_ ≈ 3 mm/s using a linear motor (Texture Analyzer TA.XTplus, Stable Micro System) and creates a fluid thread. The black area at the bottom of (**b**) indicates the fluid reservoir. Bars on the left and right of both panels represent a length of 10 cm.

The progressive thinning of free complex fluid filaments in uniaxial extension is driven by capillarity and resisted by inertia as well as viscous and elastic stresses inside the filament. Experimental techniques, such as the capillary breakup elongational rheometer (CaBER), are commonly used to study fluid response to elongational deformations. During uniaxial extension, polymer solutions and other viscoelastic fluids exhibit slow thinning processes and prolonged filament lifetimes up to several seconds, due to stretching of polymer chains and growth of extensional stresses inside the filament. On the other hand, low viscosity inelastic fluids such as water do not form long living filaments - instead the fluid thread quickly breaks. However, in this study we report a surprising phenomenon, as shown in Fig. 1, for very dilute surfactant solutions with a low shear viscosity close to water and no measurable viscoelasticity (see Supplementary Information Figure S1). This solution forms remarkably long filaments when (a) a fluid drop drips from a nozzle, and when (b) pulling a rod out of a fluid reservoir (see Supplementary Information Movie S1). Filaments with length greater than 10 cm can be created and it should be emphasized that this is not due to a viscoelastic response of the solution, which is responsible for filament formation in highly concentrated surfactant or polymer solutions. Although many studies investigated the capillary thinning of viscoelastic, shear-thinning surfactant solutions^4–8^, only two examined the uniaxial flow behavior of inelastic, shear-thickening surfactant solutions^9,10^. They reported an unexpectedly slow thinning process during CaBER measurements, where fluid threads exhibit unusually long filament lifetimes on the order of several minutes - another striking feature of the flow behavior of dilute surfactant solutions in extension. Here, we provide the first detailed explanation for this highly unexpected behavior in CaBER measurments.

From CaBER experiments, fundamental fluid properties such as the extensional viscosity and extensional relaxation time are derived based on the assumption of homogeneous elongation. However, flow instabilities can change flow behavior dramatically, and are ubiquitous during deformation of a wide variety of complex fluids including granular matter, suspensions, emulsions, foams, polymer solutions and melts, as well as aqueous surfactant solutions^11–15^. In particular, flow instabilities of surfactant solutions in shear have been extensively studied^16,17^, since these systems are widely used as additives in home and personal care products^18^, as drag reducing agents^19,20^ or as fracture fluids in enhanced oil and gas recovery^21,22^. Nevertheless, little is known about such phenomena in extension, despite the omnipresence of elongational flow fields in nature as well as in many technical processes and applications. Here, we want to understand how low viscosity fluids, such as the sample shown in Fig. 1, can form unusually long filament lengths and lifetimes, orders of magnitude larger than expected according to their shear viscosity. The type of flow instability during uniaxial elongation observed for this model system turned out to occur also in biopolymeric solutions such as saliva and hagfish slime.

## Results

### Rheology and capillary breakup of dilute surfactant solutions

**Figure 2:**
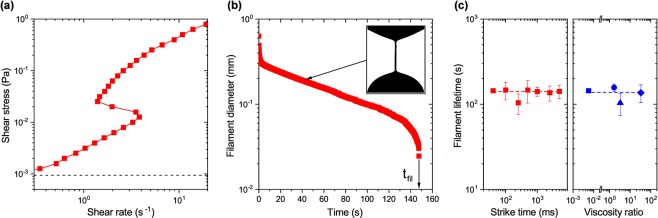
Flow properties of dilute surfactant solutions in shear and elongation. Data shown for a dilute 10 mM CTAB/NaSal, R = 0.5 surfactant solution. (**a**) Steady shear data, showing a hysteretic flow curve under controlled shear stress conditions. The dashed line represents the minimum torque limit of the rheometer. (**b**) Filament thinning behavior during capillary breakup, as probed by CaBER with initial and final height of *h*_*i*_ = 1.5 mm and *h*_*f*_ = 6 mm, respectively and strike time *t*_*s*_ = 40 ms. The inset picture shows a snapshot of the cylindrical filament at 40 s. (**c**) Filament lifetime as a function of strike time (left) and viscosity ratio between outer phase (i.e., air or silicone oil) and surfactant solution (right) in CaBER experiments. The blue square represents the filament lifetime in air as the surrounding phase.

In solutions, surfactant molecules can self-assemble into various morphologies, depending on the surfactant type and concentration, temperature and salinity^16,17^. Dilute surfactant solutions, such as the commonly used system of CTAB/NaSal mixture, [CTAB] = 10 mM, R = [NaSal/CTAB] = 0.5, shown in Fig. 1, consist of short, rigid, rodlike micellar aggregates^23^. In classical shear rheometry, this solution exhibits a non-monotonic, shear-thickening flow curve (Fig. 2(a)). At small shear rates, the shear stress increases linearly with increasing shear rate, indicating Newtonian behavior with a zero shear viscosity of *η*_0_ ≈ 3 mPa s. At a critical shear rate, a re-entrant behavior with an S-shaped flow curve is observed under controlled shear stress conditions and the viscosity increases by more than one order of magnitude. Subsequently, the viscosity passes through a maximum, followed by shear-thinning behavior.

This shear-thickening behavior is attributed to the formation of a more viscous, shear-induced state that was termed shear-induced structure (SIS)^24^. The formation of SIS can lead to banding instabilities in shear flow, where a homogeneous flow becomes unstable and splits above a critical shear rate into multiple coexisting bands, bearing different apparent viscosities and different internal structures^11,25^. In the hysteretic regime of the flow curve of surfactant solutions, multiple shear stresses coexist in the underlying constitutive curve at one imposed shear rate^11,26–28^. Turbid and transparent bands are stacked along the vorticity direction^29–31^, i.e. the axial direction in the Couette cell. Consequently this transition is referred to as vorticity banding, which was also observed for crystallizing colloids, dispersions of semi-rigid, rodlike colloids as well as nanotube suspensions^27^.

Although such banding instabilities in surfactant solutions were comprehensively studied in shear flow both experimentally and theoretically, and are well understood on the basis of continuum mechanical constitutive equations, knowledge about corresponding instabilities during uniaxial elongation is still missing, since experiments are often more challenging due to the complex filament shape and the transient nature of fluid filaments. Furthermore, most studies only investigated the vorticity banding instability of viscoelastic, shear-thinning surfactant solutions at high concentrations^29–32^. Here, we examine the flow behavior of dilute, shear-thickening surfactant solutions, focusing on the 10 mM CTAB/NaSal, R = 0.5 solution shown in Fig. 2(a). A large variety of investigated surfactant systems show similar properties in shear and elongational flow.

Surfactant solution properties in uniaxial extension are probed using a capillary breakup elongational rheometer, where the diameter of the unstable fluid thread is monitored as a function of time and the filament lifetime *t*_fil_ is determined, when the filament ruptures. Figure 2(b) shows the typical thinning behavior of a dilute surfactant solution in uniaxial elongation. At the start of the experiment, the filament thins rapidly up to a diameter of roughly 0.3 mm, forming a cylindrical thread (see insert in Fig. 2(b)). This initial fast thinning is followed by a slow thinning period until the filament ruptures at the filament lifetime. Although this 10 mM CTAB/NaSal, R = 0.5 solution exhibits a low shear viscosity of merely *η*_0_ ≈ 3 mPa s, with no apparent elasticity as probed by small amplitude oscillatory shear measurements, the sample shows a remarkably long filament lifetime of *t*_fil_ ≈ 150 s in uniaxial extension. This observation is highly unusual since the filament lifetime is more than five orders of magnitude larger than expected, assuming Newtonian flow behavior with a similar shear viscosity^33,34^.

Stretching conditions during the initial step stretch in CaBER experiments can influence filament thinning and hence the filament lifetime^8^. Further, the surface or interfacial tension between the fluid sample and the surrounding phase acts as a dominant thinning mechanism in capillary-driven breakup experiments^35^. Here, we perform CaBER experiments in different Newtonian polydimethylsiloxane (PDMS) oils as surrounding phases (AK5 *η*_0_ ≈ 5 mPa s, AK10 *η*_0_ ≈ 10 mPa s and AK100 *η*_0_ ≈ 100 mPa s), resulting in a broad range of viscosity ratios between PDMS oil and surfactant solution ($1.67\le {\eta }_{0}^{{\rm{oil}}}/{\eta }_{0}^{{\rm{surf}}}\le 33.3$). The interfacial tension between surfactant solution and air is Γ = 32.80 ± 0.01 mN/m and between surfactant solution and PDMS oil Γ = 3.84 ± 0.49 mN/m. As shown in Fig. 2(c), we find that the filament lifetime in CaBER experiments depends on neither the strike time, i.e. the time required to create the capillary bridge in CaBER experiments, nor the surrounding phase; another highly unexpected feature.

Such long lasting filaments of low concentrated surfactant solutions were reported previously by Sachsenheimer *et al*.^9^ as well as by Omidvar *et al*.^10^, and were attributed to the formation of elongation-induced structures (EIS). Based on bulk rheological elongation and shear data, the authors hypothesized that small cylindrical micelles aggregated into bigger wormlike micelles during uniaxial deformation, forming a viscoelastic network during uniaxial extension, which would lead to increased filament lifetimes. However, direct evidence of EIS buildup in uniaxial elongational of dilute surfactant solutions is still missing.

### Revealing the flow inside thinning surfactant filaments

**Figure 3:**
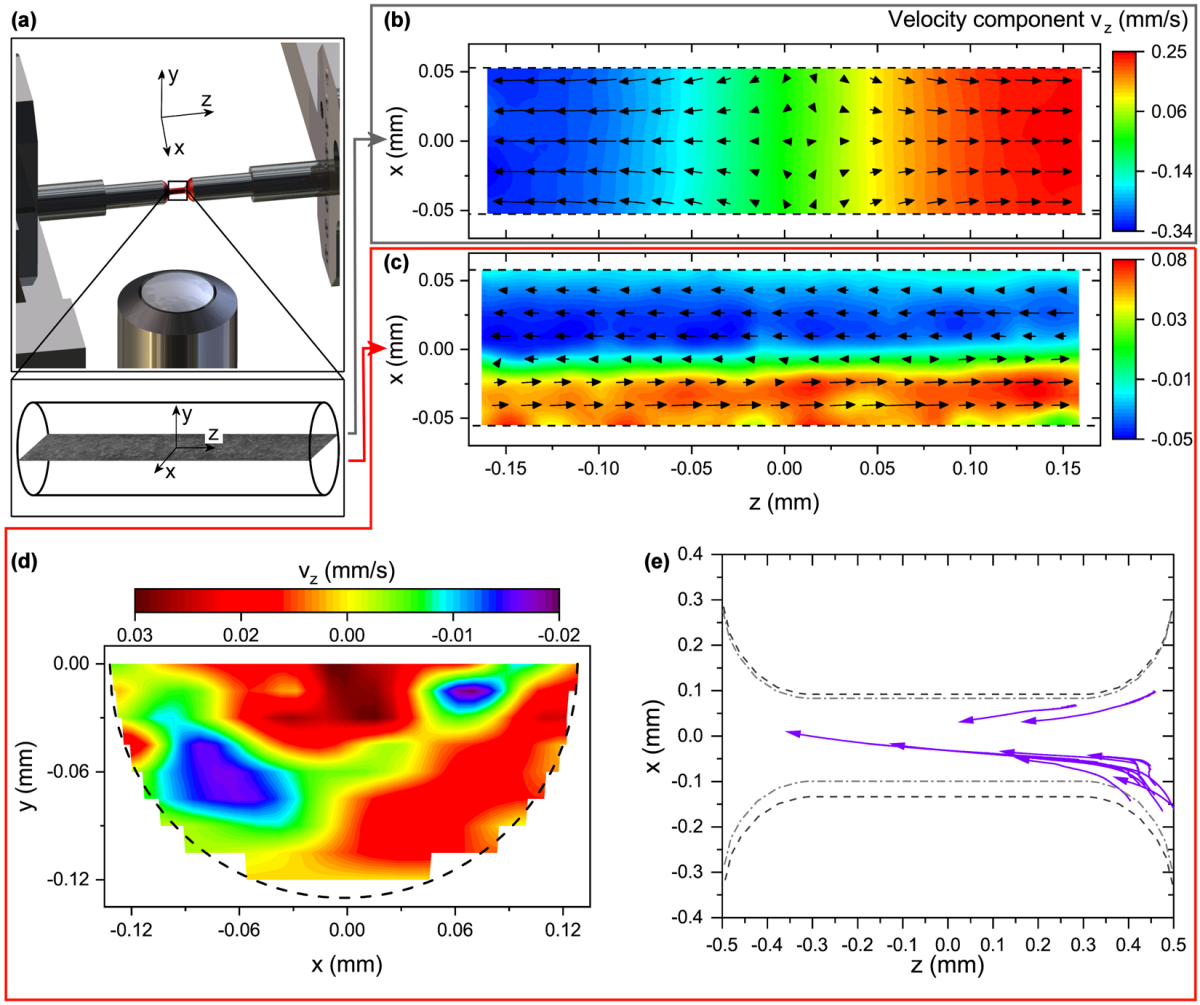
Revealing the flow inside surfactant filaments. (**a**) PIV setup for the investigation of horizontal fluid filaments. Schematic illustration of the middle part of the stretching device. The sample (red) is seeded with fluorescent particles and is placed between two circular plates that reached their final position. The lens is focused in the center between both plates and in the mid plane of the thinning filament. (**b**) Homogeneous flow for viscoelastic fluids. Velocity component in axial filament direction *v*_*z*_ inside a viscoelastic 30 mM CTAB/NaSal, R = 7.67 surfactant filament. (**c**–**e**) Heterogeneous flow inside a dilute 10 mM CTAB/NaSal, R = 0.5 surfactant filament. (**c**) Velocity component in axial filament direction *v*_*z*_, with initial displacement, final displacement, and strike time *h*_*i*_ = 1 mm, *h*_*f*_ = 4 mm, and *t*_*s*_ = 0.5 s, respectively. Dashed black lines represent the filament boundaries in the measured plane. (**d**) Axial velocity component in the lower half of a circular 10 mM CTAB/NaSal, R = 0.5 filament cross-section in the *x* − *y* plane. Note that in (**b**–**d**) the field of view is much smaller than the filament length. (**e**) Trajectories of individual tracer particles during thinning over a 20 s time interval inside a 10 mM CTAB/NaSal, R = 0.5 filament. Arrowheads indicate movement directions of the tracers. Dark grey dashed line and light grey dashed-dotted line represent the filament boundaries at *t* = 0 s and *t* = 20 s, respectively. A 10× lens and an initial *h*_*i*_ = 0.5 mm and final displacement of *h*_*f*_ = 1.5 mm are used for this experiment, hence the length of the cylindrical filament between the reservoirs is approximately *h*_*f*_ − *h*_*i*_ = 1 mm.

The experiments illustrated in Figs 1 and 2 clearly demonstrate the unique flow behavior of dilute surfactant solutions in uniaxial extension. In an effort to reveal the origin of the long filament lifetime, we visualize the flow inside horizontally stretched surfactant threads. We perform quantitative measurements of the flow field inside thinning surfactant filaments by employing particle image velocimetry (PIV). A customized stretching device (Fig. 3(a)), similar to CaBER, is used to create horizontal filaments, seeded with fluorescent microspheres. Images are recorded throughout the whole thinning process, making a longitudinal cut through the mid plane of the center part of the slender filament.

Figure 3(b) shows the velocity component in axial filament direction *v*_*z*_ as spatial color plot representation with superimposed velocity vectors for a standard viscoelastic, shear-thinning 30 mM CTAB/NaSal, R = 7.67 solution at the beginning of the thinning process (*t*/*t*_fil_ = 1/4). Note, that here the field of view (350 × 350 *μ*m) is much smaller than the filament length, i.e. *h*_*f*_ − *h*_*i*_ = 3 mm, thus only flow in the center of the fluid thread is examined. For a viscoelastic solution, the flow field during uniaxial elongation is homogeneous, with flow in both negative and positive axial direction, axisymmetric to the centrally located stagnation point. This homogeneous flow is expected for capillary thinning of viscoelastic filaments in uniaxial extension^36^. Further, we find that the velocity component in x direction *v*_*x*_ is always more than one order of magnitude smaller than *v*_*z*_, in agreement with Gier and Wagner^36^. Hence, *v*_*x*_ will not be considered in the following. In contrast to the dilute surfactant solutions discussed above, the 30 mM CTAB/NaSal, R = 7.67 sample shown in Fig. 3(b) exhibits a Newtonian plateau with a low-shear viscosity of *η*_0_ ≈ 18 Pa s, followed by a shear-thinning regime.

For the dilute, shear-thickening 10 mM CTAB/NaSal, R = 0.5 solution (Fig. 3(c)), which exhibits long filaments and long filament lifetimes (Figs 1 and 2), an unexpected flow behavior emerges. We find flow in both positive and negative axial direction across the filament diameter, indicated by the superimposed velocity vectors at *t*/*t*_fil_ = 1/4. During filament thinning, two bands form inside the filament mid plane, transporting fluid in opposing axial directions, in striking contrast to the expected flow kinematics shown in Fig. 3(b). This heterogeneous flow pattern persists throughout the whole thinning process, but the shape of the flow profile and the magnitude of *v*_*x*_ change over time (see Supplementary Information Figure S2). The band with flow in positive axial direction broadens, while the width of the other band seems to decrease until only flow in one direction leads to filament breakup. This is the first observation of such a heterogeneous banding phenomenon during thinning of a fluid in uniaxial elongational deformation. The non-uniform velocity pattern results in a velocity gradient, hence a shear rate distribution $\dot{\gamma }(x)$ across the filament diameter, with regions of localized high shear rate between different bands.

Performing multiple experiments with the dilute surfactant solution always yields heterogeneous flow patterns, yet the number of bands and the magnitude of *v*_*z*_ in the bands seems to change arbitrarily from measurement to measurement. Furthermore, this heterogeneous flow occurs irrespective of stretching conditions or the surrounding phase, as long as conditions are such that cylindrical filaments form. For the higher concentrated surfactant/salt viscoelastic sample, velocity profiles show a homogeneous, plug-like flow that persists throughout the whole thinning process until the filament breaks (see Supplementary Information Figure S2). Here, the velocity gradient across the filament diameter is negligible within experimental error.

Furthermore, we find that for dilute surfactant samples, the flow in the whole filament cross-section is heterogeneous, and not only in the middle of the cylindrical filament, as shown in Fig. 3(b). We use a long travel range objective scanner to capture the flow inside the circular filament cross-section. Figure 3(d) shows the velocity component in axial filament direction in the *x* − *y* plane of the lower half of a 10 mM CTAB/NaSal, R = 0.5 filament. From this representation, it is clear that the heterogeneous flow exists in the whole cross-section without any rotational symmetry. At one fixed *x* position, e.g. *x* = −0.06 mm, flow direction changes along the filament depth *y*. Note that since the heterogeneous flow is not rotationally symmetric, we use Cartesian coordinates in Fig. 3. The fibrillar, banded flow seems to occur primarily in the flow-vorticity-plane, as indicated in Fig. 3(c), similar to the banded flow in a Couette cell in steady shear experiments.

In our experiments shown in Fig. 3(b–d), the field of view (350 × 350 *μ*m) is much smaller than the filament length (roughly 3 mm), thus the heterogeneous flow is only visible in the central part of the filament. In Fig. 3(e), we decrease the final length (1.5 mm) and use a lower magnification lens to elucidate the flow field in the whole filament between the reservoirs. Here, we track individual tracer particles that are in the right-hand reservoir at *t* = 0 s over a 20 s time interval. Our results show that particles travel from one reservoir across the filament into the opposite reservoir. This means that the heterogeneous flow exists across the whole filament length and leads to an additional flow from the reservoirs into the filament besides the expected surface tension driven flow from the filament into the reservoirs at each end. A circulating flow merely inside the filament is not observed. Trajectories that seem to start inside the filament arise from tracers entering the focal plane during data acquisition.

**Figure 4:**
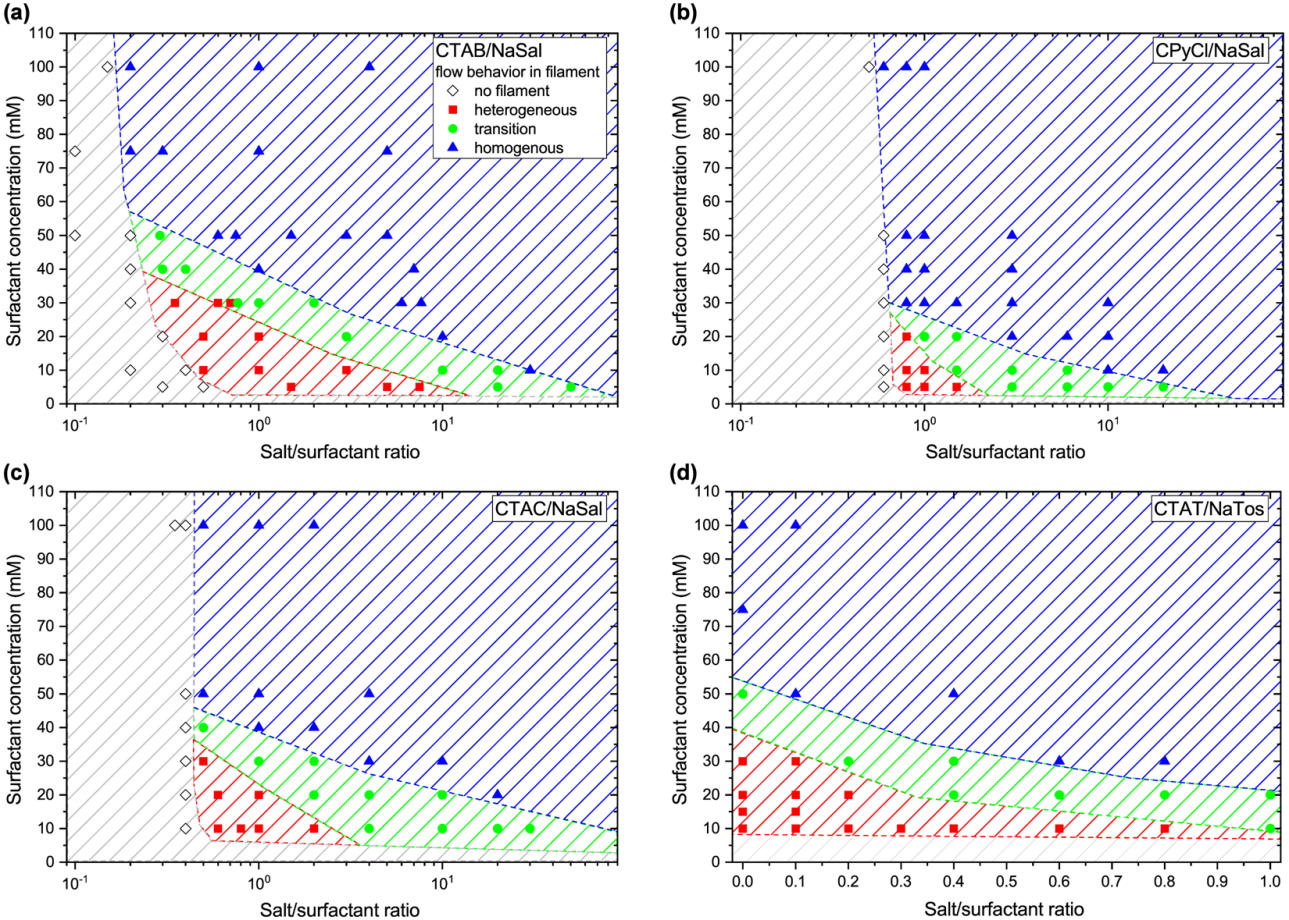
Flow behavior phase diagrams of common surfactant systems. (**a**) CTAB/NaSal, (**b**) CPyCl/NaSal, (**c**) CTAC/NaSal, (**d**) CTAT/NaTos. Hatched areas are to guide the eye.

In Fig. 4, we summarize our results regarding the flow behavior inside surfactant filaments for four commonly investigated surfactant/salt systems: (a) CTAB/NaSal, (b) CPyCl/NaSal, (c) CTAC/NaSal and (d) CTAT/NaTos. All systems show similar features in the investigated concentration regimes. If the surfactant concentration or the amount of salt is too low, e.g. in Fig. 4(a) for surfactant concentrations smaller than 5 mM or salt/surfactant ratios smaller than 0.2, no filaments form during uniaxial deformation. When filaments can be created, we find three types of flow behavior. For low surfactant concentrations, the flow is heterogeneous with flow in both axial directions (cf. Fig. 3(c)). This heterogeneous flow leads to the additional flow from the reservoirs into the filament, thus increasing the filament lifetime. In contrast, for high surfactant concentration and salt/surfactant ratio, a homogeneous, ideal uniaxial flow emerges (cf. Fig. 3(b)). Between the heterogeneous and homogeneous domains, a transition regime appears. Here, we find either homogeneous or heterogeneous flow while performing multiple experiments. The extent of the unstable flow regimes varies between the different surfactant systems, and is greatest for CTAB/NaSal (red area in Fig. 4(a)). For CTAT/NaTos, filaments can be created even without the addition of salt and we therefore plot the *x* axis linearly. This can be explained as follows. For the samples in Fig. 4(a–c), the halogen counterions *Br*^−^ and *Cl*^−^ dissociate in water and the added salt NaSal promotes anisotropic micellar growth^16,17^. In the case of CTAT, the surfactant molecule consists of the cationic surfactant chain and the anionic molecule p-toluenesulfonate. If the pure surfactant is dissolved in water, the dissociation of p-toluenesulfonate is sufficient to promote the formation of elongated micelles without the addition of further salt. In this case, heterogeneous flow occurs even without additional salt for surfactant concentrations smaller than 40 mM.

Heterogeneous banding inside the filament is induced by extensional deformation and we demonstrated multiple flow bands with different velocities and even reversed flow direction. However, evidence of a possible corresponding microstructural transition, coupled to banding instabilities in surfactant solutions, is still missing. If the heterogeneous flow, characterized by multiple bands at one macroscopic elongation rate, arises from a flow instability, we may find critical conditions in terms of strain rate and total strain for this transition. In CaBER experiments, long lasting filaments form as soon as the fluid thins after the initial step stretch. This suggests that the banding instability arises before the fluid thread reaches its slow thinning period (see Fig. 2(b)). For the standard configuration used here, the maximum accumulated strain during the initial step stretch is *ε* ≈ 6, while the elongation rate passes through a range of $1\le \dot{\varepsilon }\le 10$ s^−1^, as derived from the diameter decay (see Supplementary Information Figure S3). These apparent values may give an idea for the critical parameters, necessary to induce the heterogeneous flow phenomenon. However, the elongation rate during the step stretch in CaBER experiments is not a constant, but specified according to stretching parameters, viscous and elastic stresses inside the tested fluid, as well as inertia effects during the early thinning. Furthermore, the cylindrical filament shape inhibits spatially resolved optical techniques, such as birefringence imaging, to probe structural changes and orientation of micelles during elongation on a microscopic length scale. To address these challenges, we use a microfluidic optimized-shape cross-slot to generate a planar extensional flow field with a controlled elongation rate. The planar flow in the deep channel enables us to make meaningful measurements of the flow-induced birefringence (FIB), which allows to track flow-induced structural changes at negligible inertia and without the influence of potential evaporation as well as surface tension.

### Time-dependent flow instability in planar elongation

The optimized-shape cross-slot extensional rheometer (OSCER) generates an almost ideal planar elongational flow field and has been used to study the flow behavior and instabilities of polymer and surfactant solutions in planar elongation^37,38^. Here, we perform flow velocimetry experiments and measurements of flow-induced birefringence of surfactant solutions in planar extension, using a microfluidic OSCER device. Further, we use dye imaging as another method to quantify time-dependent transitions of the flow field.

**Figure 5:**
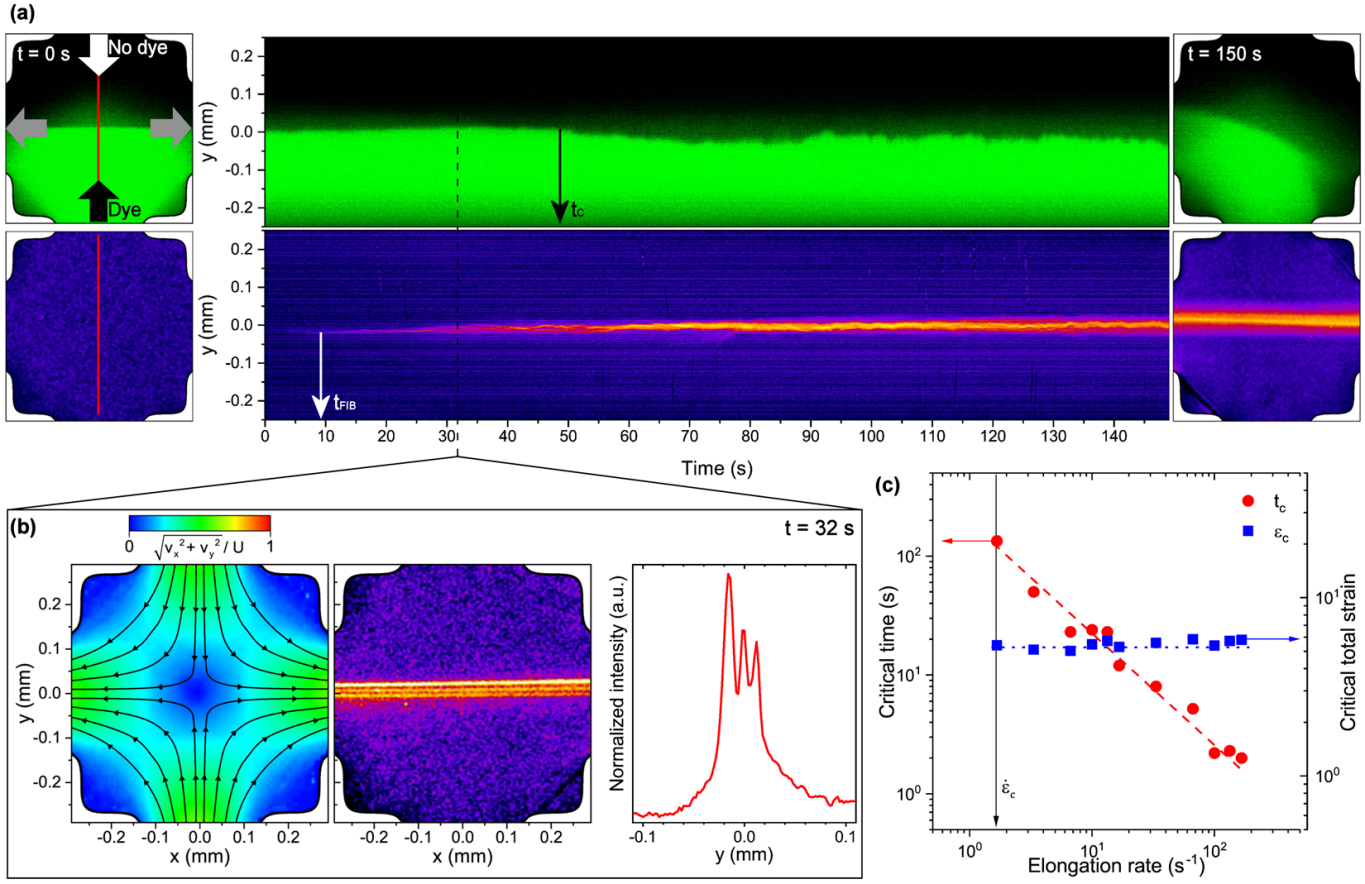
Time-dependent flow behavior of dilute surfactant solutions in planar extension. Results are shown for a 10 mM CTAB/NaSal, R = 0.5 solution in the OSCER. (**a**) Evolution of the flow field (top) as probed by dye imaging and birefringence (bottom) at an elongation rate of $\dot{\varepsilon }=3$ s^−1^ and *Re* = 0.19. (Left) Images at start of experiment *t* = 0 s, (middle) time series plot across red lines and (right) at *t* = 150 s. Colors represent normalized intensities of the dye and qualitative birefringence. (**b**) Normalized velocity (left) with superimposed hyperbolic streamlines from PIV measurements, normalized intensity of FIB in the whole OSCER device (middle) and across x = 0 (right) at *t* = 32 s after the experiment started, for the same experimental conditions as in (**a**). (**c**) Critical time required for the onset of asymmetry breaking and critical total strain as a function of the applied elongation rate.

In Fig. 5(a) we show confocal microscopy images of the dye imaging (top) and the normalized intensity for the qualitative FIB measurement (bottom) at *t* = 0 s (left) and 150 s (right), respectively. The elongation rate is $\dot{\varepsilon }=3$ s^−1^ and the Reynold number is *Re* = 0.19. Space-time diagrams (middle) show the evolution of the signals across the mid axis, indicated by the red lines at *t* = 0 s. At the beginning, the flow field stays symmetric until the flow bifurcates at a critical time *t*_*c*_, indicated by the fluctuations in the interface between the dyed and undyed solutions in the top of Fig. 5(a). These results are in quantitative agreement with time-resolved PIV measurements. Prior to the symmetry breaking, a single birefringent strand emerges when the flow is still symmetric. This strand splits into several bands that start to oscillate laterally, similar to the spatio-temporal fluctuations of the asymmetric flow field. However, the number of bands seems to change over time as the strands collapse and split continuously. We note that the exposure time in qualitative FIB measurements is much smaller than the temporal fluctuations of the FIB signal reported in Fig. 5.

To emphasize the occurrence of multiple birefringent strands, we show the velocity field and the birefringence signal in the OSCER device at *t* = 32 s in Fig. 5(b). Here, the flow field is still symmetric, with a centrally located stagnation point about which incoming hyperbolic streamlines divide symmetrically. A constant elongation rate is imposed on the fluid along the outflow direction. While the flow field is still uniform and well-defined, flow-induced birefringence emerges along the outflow direction and three individual birefringent bands are clearly distinguishable in the middle panel of Fig. 5(b). Consequently, we find bands of different stresses coexisting at the same deformation rate. The right panel of Fig. 5(b) shows the normalized birefringence intensity across the y direction at x = 0. We estimate the spatial scale of the mesoscopic bands to be 15 *μ*m, much larger than the length scale of WLM bundles (350 nm, as deduced from scanning electron microscopy (SEM)) that formed flow-induced structured phases in similar dilute surfactant solutions^39^. At higher surfactant and salt concentrations, WLM can grow up to 3 *μ*m, as derived light scattering experiments under rest^40^, as well as estimated from their zero shear rheological properties^41^. Even these length scales are much smaller than the spatial dimensions we observe in Fig. 5(b).

Employing time-resolved PIV measurements and dye imaging on dilute surfactant samples, we determine the time for the onset of the symmetry breaking instability shown in Fig. 5(a). This time decreases with increasing applied elongation rates, as shown in Fig. 5(c). Scaling the critical time with the corresponding elongation rate yields a constant critical strain of *ε*_*c*_ ≈ 5.5. We note that for elongation rates smaller than the critical rate ${\dot{\varepsilon }}_{c}=1.5$ s^−1^, the flow stays Newtonian-like with undetectable FIB signal.

### Onset of flow instabilities in different flow kinematics

Finally, we relate the results in planar and uniaxial elongation to the hysteretic flow curve from steady shear experiments. We find that the flow instability in the OSCER emerges at a critical nominal strain rate of ${\dot{\gamma }}_{\mathrm{nom},O}=2\,{\dot{\varepsilon }}_{c}\approx 3$ s^−1^. This nominal value is in the hysteretic regime of the flow curve, obtained from steady shear rheometry in Fig. 2(a). In shear, the flow separates into vorticity bands with multivalued stress, stacked along the vorticity direction, and flow-induced structures form in this shear rate regime. Similarly, we find bands of different stresses coexisting at the same deformation rate in OSCER experiments, indicated by multiple birefringent bands^42^. For CaBER measurements, the nominal strain rate varies during the initial step stretch as discussed earlier. In this case, we find ${\dot{\gamma }}_{\mathrm{nom},C}=\sqrt{3}\,{\dot{\varepsilon }}_{c}$, thus $1.7\le {\dot{\gamma }}_{\mathrm{nom},C}\le 17$ s^−1^. Furthermore, we find that the heterogeneous velocity distribution across the filament diameter results in regions of localized high shear rate between the different bands with $2\le \dot{\gamma }(x)\le 5$ s^−1^. In summary, banding instabilities in elongational flow emerge at nominal strain rates corresponding to the hysteresis regime of the non-monotonic shear flow curve, which is a necessary condition for the existence of a stationary vorticity banded state^11^. Hence, similar strain rates are required to induce banding phenomena in these systems, irrespective of flow kinematics. We therefore state that the reported phenomena occurring in elongational flows are due to “extensional vorticity banding”. In shear flow, the origin for the increases of viscosity and vorticity banding is the formation of a new and more viscous phase. In uniaxial extension, our PIV results show that structure formation during extension does not occur homogeneously on a mesoscopic length scale throughout the filament, e.g. no uniform flow-induced structured phase, with entangled, branched, and multiconnected micellar bundles is formed, as reported previously for semi-dilute wormlike micellar solutions flowing through microposts^39^. Instead, we propose that during the initial step stretch in CaBER experiments, strain rate and total strain experienced by the sample are sufficient to induce a banding instability, leading to the formation of heterogeneous structures. This heterogeneity occurs randomly in the filament. The strong coupling between internal structure and flow leads to regions of localized high shear rate between coexisting macroscopic bands, where new structures can form.

### Heterogeneous flow inside biopolymeric threads

Although we focus on the elongational flow behavior of dilute surfactant solutions as a model system in this study, preliminary results demonstrate that biopolymeric fluids such as hagfish slime and whole human saliva also show banded flow during filament thinning (see Supplementary Information Figure S4). Whole saliva is a mixture of different glandular secretions^43^ and hagfish slime contains mucin vesicles and thread skeins^44^. Both naturally heterogeneous fluids show filament lifetimes of several seconds and exhibit a heterogeneous velocity distribution inside the cylindrical thread, similar to the flow inside thinning filaments of dilute surfactant solutions. Note that PIV measurements are performed prior to the formation of beads-on-a-string structures. Figure S4 shows that the heterogeneous flow phenomenon is not limited to aqueous surfactant solutions, but is a generic phenomenon during extensional deformation of complex fluids occurring in nature or technology. Further investigations will be required to elucidate the origin for the heterogeneous flow in these fluids.

## Conclusion and Discussion

Employing flow velocimetry on horizontally stretched filaments, we discovered a unique banding phenomenon, governing the flow during breakup of liquid threads. We demonstrated that a heterogeneous distribution of the axial velocity component in the whole filament cross-section, with multiple bands of flow in opposite filament direction, results in global filament lifetimes of several minutes and remarkably long filaments of dilute surfactant solutions during uniaxial elongation. We find this heterogeneous flow behavior in the surfactant solutions investigated by Sachsenheimer *et al*.^9^ and Omidvar *et al*.^10^, who attributed the observation of such long filament lifetimes to the formation of elongation-induced structures (EIS) without further experimental evidence. Moreover, Sachsenheimer *et al*.^9^ proposed a map for the occurrence of elongation-induced structure formation in CTAB/NaSal solutions. The regime for which they proposed the formation of EIS, based on long filament lifetimes, matches the heterogeneous flow regime shown in Fig. 4(a) based on our PIV data. For the particular sample 30 mM CTAB/NaSal, R = 7.67, EIS formation was confirmed by flow-induced onset of turbidity in planar squeeze flow and planar elongation^45–47^. However, this highly viscoelastic sample shows homogeneous flow behavior in uniaxial extension, as demonstrated by our PIV experiments. The dilute surfactant systems also exhibit a flow instability in planar extension, with the emergence of multiple birefringent strands, indicating multiple stresses at one global rate. This is the first experimental observation of such a multivalued relation between stress and deformation rate in extension that was recently predicted using a mechanistic constitutive model^48^. Although we are not able to resolve the full mechanism of flow-induced structure formation in this complex scenario up to now, our experiments in elongation as well as in shear enable us to reveal a new banding instability in elongational flows and demonstrate that the occurrence of a flow instability in such solutions is a generic phenomenon, independent of flow kinematics. Our experimental method can be readily extended to study the flow inside other low viscosity fluid filaments in extensional flow fields, ubiquitous in nature or technology, and preliminary results show that biopolymeric fluids such as saliva and hagfish slime also exhibit banded flow during filament thinning.

## Materials and Methods

### Sample preparation

Aqueous surfactant solutions of four commonly studied cationic surfactants hexadecyltrimethylammonium bromide (CTAB), hexadecylcetylpyridinium chloride (CPyCl), hexadecyltrimethylammonium chloride (CTAC) and hexadecyltrimethylammonium p-toluenesulfonate (CTAT) were studied. The salt sodium salicylate (NaSal) was added to solutions containing CTAB, CPyCl and CTAC, to enhance the anisotropic growth of micelles. Samples of CTAT were prepared with the addition of the salt sodium p-toluenesulfonate (NaTos). Materials were purchased as powders (Sigma Aldrich and Carl Roth) and were used without further purification. Aqueous solutions were prepared by dispersing weighed quantities of surfactant and salt in ultrapure water. Samples were shaken thoroughly for five days to assist dissolution. Subsequently, the solutions were stored at 20 °C for one week to ensure the formation of equilibrium micellar structures.

### Shear rheological characterization

Steady shear experiments were performed using a stress controlled rotational rheometer (MCR 501, Anton Paar), equipped with a coaxial cylinder fixture (CC27, inner radius *R*_*i*_ = 13.330 mm, radii ratio *δ* = *R*_*a*_/*R*_*i*_ = 1.0849). We measured in controlled rate and controlled stress mode, increasing or decreasing the applied shear rate or shear stress, respectively. This allowed for investigation of possible hysteresis phenomena in the shear-thickening regime of the flow curve. All measurements were performed at 20 °C.

We performed small-amplitude oscillatory shear (SAOS) experiments, using a strain amplitude of *γ*_0_ = 1% and determined the viscoelastic properties of the dilute surfactant solutions in the frequency range of $0.1\le \omega \le 50$ rad/s. Passive microrheology was employed by seeding the dilute surfactant solutions with 0.01 wt% of mono-disperse fluorescent polystyrene microspheres (Banglabs Inc.) with a diameter *d*_*p*_ = 0.96 *μ*m. The storage and loss moduli were calculated from the mean square displacement of the embedded particles, based on the generalized Stokes-Einstein relation^49^.

### Capillary thinning experiments

Surfactant solution properties in uniaxial extension were probed using a commercial capillary breakup extensional rheometer (CaBER, Thermo Scientific). The setup was extended with an optical train, consisting of a high-speed camera (Fastcam-X 1024 PCI, Photron), a telecentric objective (TC4M 16, 1×, MaxxVision) and a blue telecentric back light illumination (TZb30, Vision & Control). Choosing plates with a diameter *D*_0_ = 6 mm, an initial height *h*_*i*_ = 1.5 mm and final height of *h*_*f*_ = 6 mm, allowed to characterize the thinning process of surfactant solutions for a broad range of surfactant and salt concentrations.

We performed CaBER experiments in different oils as surrounding phases, using a customized transparent cell that enclosed the CaBER plates and the fluid sample. The cell was filled with Newtonian polydimethylsiloxane (PDMS) oils with different viscosities (AK5 *η*_0_ ≈ 5 mPa s, AK10 *η*_0_ ≈ 10 mPa s and AK100 *η*_0_ ≈ 100 mPa s, Wacker Chemie AK), resulting in a broad range of viscosity ratios between the PDMS oil and the surfactant solution. Interfacial tensions were measured using a drop shape analyzer (DSA100, Krüss). The interfacial tension between surfactant solution and air was Γ = 32.80 ± 0.01 mN/m and between surfactant solution and PDMS Γ = 3.84 ± 0.49 mN/m, independent of surfactant and PDMS oil type, as well as surfactant and salt concentration.

### Flow visualization in uniaxial elongation during horizontal filament thinning

We performed quantitative measurements of the flow field inside thinning surfactant filaments by employing particle image velocimetry (PIV). Therefore, samples were seeded with 0.01 wt% of mono-disperse fluorescent polystyrene microspheres (Banglabs Inc.) of diameter *d*_*p*_ = 0.96 *μ*m with excitation and emission wavelengths of 480 nm and 520 nm, respectively. A customized stretching device (Fig. 3(a)), similar to CaBER, was used to create horizontal filaments between two circular plates with diameter *D*_0_. One plate was fixed, while the other plate was separated from an initial length *h*_*i*_ to a final length *h*_*f*_ within a time *t*_*s*_ to create a horizontal filament of length (*h*_*f*_ − *h*_*i*_). If not mentioned otherwise, we used *D*_0_ = 4 mm, *h*_*i*_ = 1 mm, *h*_*f*_ = 4 mm and *t*_*s*_ = 0.5 s as stretching parameters. The device was mounted on an inverted fluorescence microscope (AxioObserver D, Carl Zeiss AG) equipped with an sCMOS Zyla X camera (Andor Technology, 2048 × 2048 pixels, up to 50 fps) and a long working distance lens (Nikon LU-Plan, 20×, NA = 0.4). A constant LED light source (Colibri, 470 nm, Carl Zeiss AG) was used for volume illumination of the cylindrical filament. With this configuration, the PIV measurement depth over which particles contribute to the determination of the flow field was *δz*_*m*_ ≈ 15 *μ*m^50^.

After the plate reached the final displacement, images were recorded throughout the whole thinning process, making a longitudinal cut through the mid plane of the center part of the slender filament. Particle images were captured and analyzed in pairs, where the time delay between two images in each pair was chosen such that particle displacement was around 4 pixels. We used an open source PIV software^51^ for quantitative analysis of the flow field. Velocity vectors were obtained in interrogation areas of 32 × 32 pixels in *x* and *z* direction.

Furthermore, we used a long travel range objective scanner (P-a 725.4CD, Physik Instrumente (PI) GmbH & Co. KG) to capture the three-dimensional (3D) flow inside the lower half of the circular filament. Image pairs in the *x* − *z* plane were recorded in ten slices, with spacing in depth of the filament matching the measurement depth of the PIV setup. The total number of image pairs was recorded over a time of roughly 1 s that was much shorter than the filament lifetime, hence thinning in radial direction and spatial shifting of the velocity bands could be neglected. PIV analysis was performed in each slice and the velocity component in axial filament direction was extracted at the same axial position.

For particle tracking, we used a custom particle tracking routine incorporated into the software Image Processing System (Visiometrics iPS) and a self-written Matlab program based on the widely used Crocker and Grier tracking algorithm^52^.

### Flow in planar elongation using a microfluidic cross-slot

We performed flow velocimetry experiments and measurements of flow-induced birefringence (FIB) of surfactant solutions in planar extension, using a microfluidic OSCER device. The channel was fabricated via selective laser-induced etching^53,54^ in bulk fused silica, utilizing a LightFab 3D printer (LightFab GmbH). The cross-slot was fitted between two glass windows to enclose the channels with optical access. Four holes were ultrasonically drilled in one window to connect the channel with in- and outlet tubing. We used four servo-driven syringe pumps (neMESYS, Cetoni GmbH), fitted with Hamilton Gastight syringes, to inject fluid in two opposed inlet channels and simultaneously withdraw fluid from the other two opposed outlet channels at equal rates. The depth of the OSCER device was *d* = 2 mm and the width of the in- and outlet channels were *w* = 100 *μ*m, resulting in a high aspect ratio of *d*/*w* = 20. The Reynolds number *Re* in the OSCER was defined as *Re* = *ρUD*_*h*_/*η*_0_, where *ρ* and *η*_0_ are the fluid density and zero shear viscosity, respectively. *U* = *Q*/(*wd*) is the average flow velocity and *D*_*h*_ = 2*wd*/(*w* + *d*) is the hydraulic diameter. For the shear-thickening sample 10 mM CTAB/NaSal, R = 0.5 discussed above, we use *ρ* = 0.996 g/mL and *η*_0_ = 3 mPa s to calculate *Re*.

The flow field inside the OSCER in the *x* − *y* plane (*z* = 0) was characterized using a *μ* PIV system (TSI Inc.). Samples were seeded with 0.02 wt% carboxylate-modified microspheres (FluoSpheres) of diameter *d*_*p*_ = 1 *μ*m with excitation and emission wavelengths of 535 nm and 575 nm, respectively. The OSCER device was mounted on an inverted microscope (Nikon Eclipse Ti), equipped with a CMOS camera (1280 × 800 pixels, Phantom Miro M310, Vision Research Inc.) and a 10× lens (Nikon Plan Fluor, NA = 0.3). The measurement depth with this setup was *δz*_*m*_ ≈ 30 *μ*m. The camera was synchronized with a dual-pulsed Nd:YLF laser (Terra PIV, Continuum Inc.), which illuminates the sample with short laser pulses. A standard cross-correlation PIV algorithm (TSI Insight 4 G software), with interrogation areas of 32 × 32 pixels and Nyquist criterion was used to perform quantitative analysis of the flow field inside the microfluidic device. For a Newtonian fluid, steady flow behavior in the OSCER device is characterized by a flow field with a centrally located stagnation point about which hyperbolic streamlines divide symmetrically. Here, the velocity in *x* direction along *y* = 0 is proportional to *x*, hence the velocity gradient $\partial {v}_{x}/\partial x{|}_{y=0}$ is constant and defines the elongation rate $\dot{\varepsilon }$ imposed on the fluid in the OSCER. For our channel geometry, we found the proportionality $\dot{\varepsilon }=\mathrm{0.197\ }U/w$, in close agreement with the expectation for a two-dimensional flow, indicating that the flow profile was almost uniform through most of the depth.

Additionally, we used dye imaging to quantify the time at which the flow undergoes transition. We employed a differential spinning disk (DSD2) confocal microscope (Andor Technology Ltd) and used a surfactant solution containing 10 *μ*M fluorescent rhodamine B flowing through one inlet, while an undyed solution was introduced in the second inlet channel.

We visualized time-resolved qualitative FIB inside the OSCER using an optical train that consisted of a white light source, a polarizer (45°), the flow cell with outlets oriented at 0°, a second polarizer (−45°) and a CCD camera (1025 × 1024 pixels) with a 10× lens. Using this configuration, the measured intensity depended upon the micellar orientation angle as well as the degree of micellar alignment. However, since in the OSCER device the orientation is known to be along the stretching direction (i.e. 0°), the signal intensity was related to the optical anisotropy, with bright regions indicating more significant alignment of micelles. Based on the stress-optical rule, the signal intensity is also related to the principal stress difference in the flowing fluid^42^. Images were recorded with an exposure time of 20 ms at 10 Hz.

## Supplementary information

Movie S1

Supplementary Information

## Data Availability

All data needed to evaluate the conclusions in the paper are present in the paper and the Supplementary Materials. Additional data related to this paper may be requested from the authors.
