# Supplementary material for: Heterogeneous flow inside threads of low viscosity fluids leads to anomalous long filament lifetimes
Source: Sci Rep. 2019 May 8;9:7110. doi: 10.1038/s41598-019-43590-z (PMC6506508; doi:10.1038/s41598-019-43590-z)
Supplement: Supplementary file 2 — Supplementary Information [file 41598_2019_43590_MOESM2_ESM.pdf]

Supplementary Information for "Heterogeneous flow inside threads of low viscosity fluids leads to anomalous long filament lifetimes"

Steffen M. Recktenwald[1,*], Simon J. Haward[2], Amy Q. Shen[2], and Norbert Willenbacher[1]

[1]Karlsruhe Institute of Technology, Institute for Mechanical Process Engineering and Mechanics, Gotthard-Franz-Straße 3, 76131 Karlsruhe, Germany
[2]Okinawa Institute of Science and Technology Graduate University, Micro/Nano/Biofluidics Unit, 1919-1 Tancha, Onna-son, Okinawa 904-0495, Japan
[*]steffen.recktenwald@kit.edu

Supplementary Movie

- Movie S1: Real time video of filament stretching experiments. Filament formation using a dyed 10 mM CTAB/NaSal, R = 0.5 surfactant solution.

Supplementary Figures

- Figure S1: Dilute surfactant solutions show no measurable elasticity.
- Figure S2: Evolution of flow profiles over time inside surfactant filaments.
- Figure S3: Capillary thinning of dilute surfactant solutions.
- Figure S4: Heterogeneous flow inside thinning biopolymer filaments.

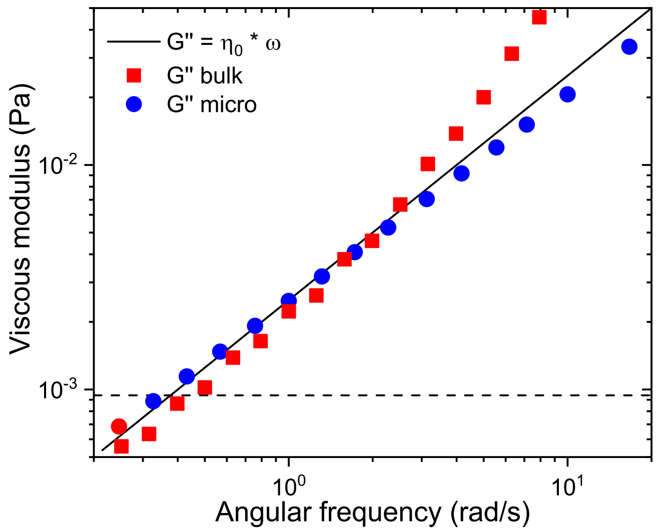

Figure S1. Dilute surfactant solutions show no measurable elasticity. Viscous modulus G″ of a 10 mM CTAB/NaSal, R = 0.5 solution determined through bulk small-amplitude oscillatory shear (SAOS) measurements (red squares) as well as using passive microrheology (blue circles). The black dashed line indicates the minimum torque limit of the rheometer and the solid black line represents the viscous properties of the solution, calculated from the zero shear viscosity. At higher frequencies G″ derived from SAOS measurements deviates from the microrheological measurement and the apparent modulus increases close to $G'' \sim \omega^2$, indicating the onset of instrument-inertia effect in bulk measurements. The elastic modulus G′ could not be determined.

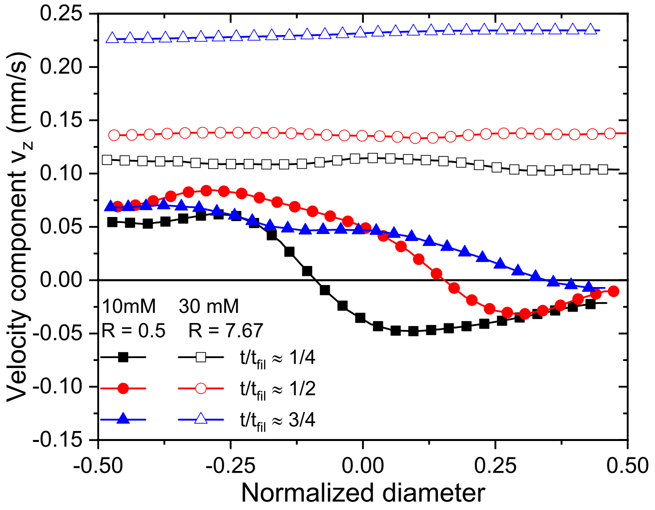

Figure S2. Evolution of flow profiles over time inside surfactant filaments. Flow profiles of the axial velocity component v_z over the normalized filament diameter for a 10 mM CTAB/NaSal, R = 0.5 solution (closed symbols) and a 30 mM CTAB/NaSal R = 7.67 solution (open symbols).

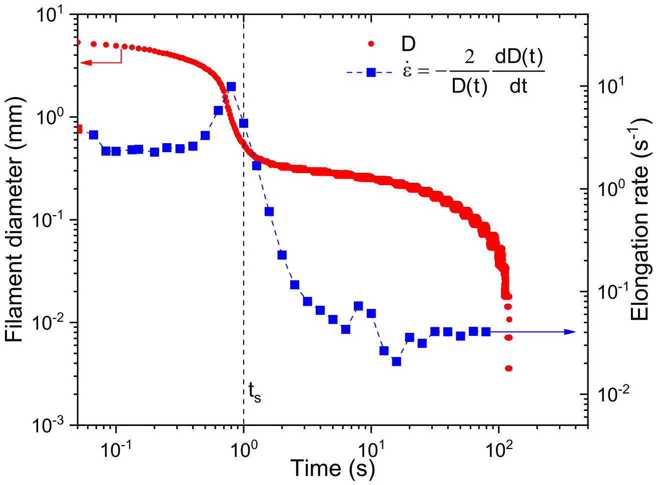

Figure S3. Capillary thinning of dilute surfactant solutions. Diameter as a function of time (red circles) for a 10 mM CTAB/NaSal, R = 0.5 filament during step stretch and subsequent thinning, using CaBER. Stretching parameters are $D_0 = 6$ mm, $h_i = 1.5$ mm, $h_f = 6$ mm, and $t_s = 1$ s. Blue squares display the local derivative of $D(t)$, hence the local elongation rate $\dot{\varepsilon}$.

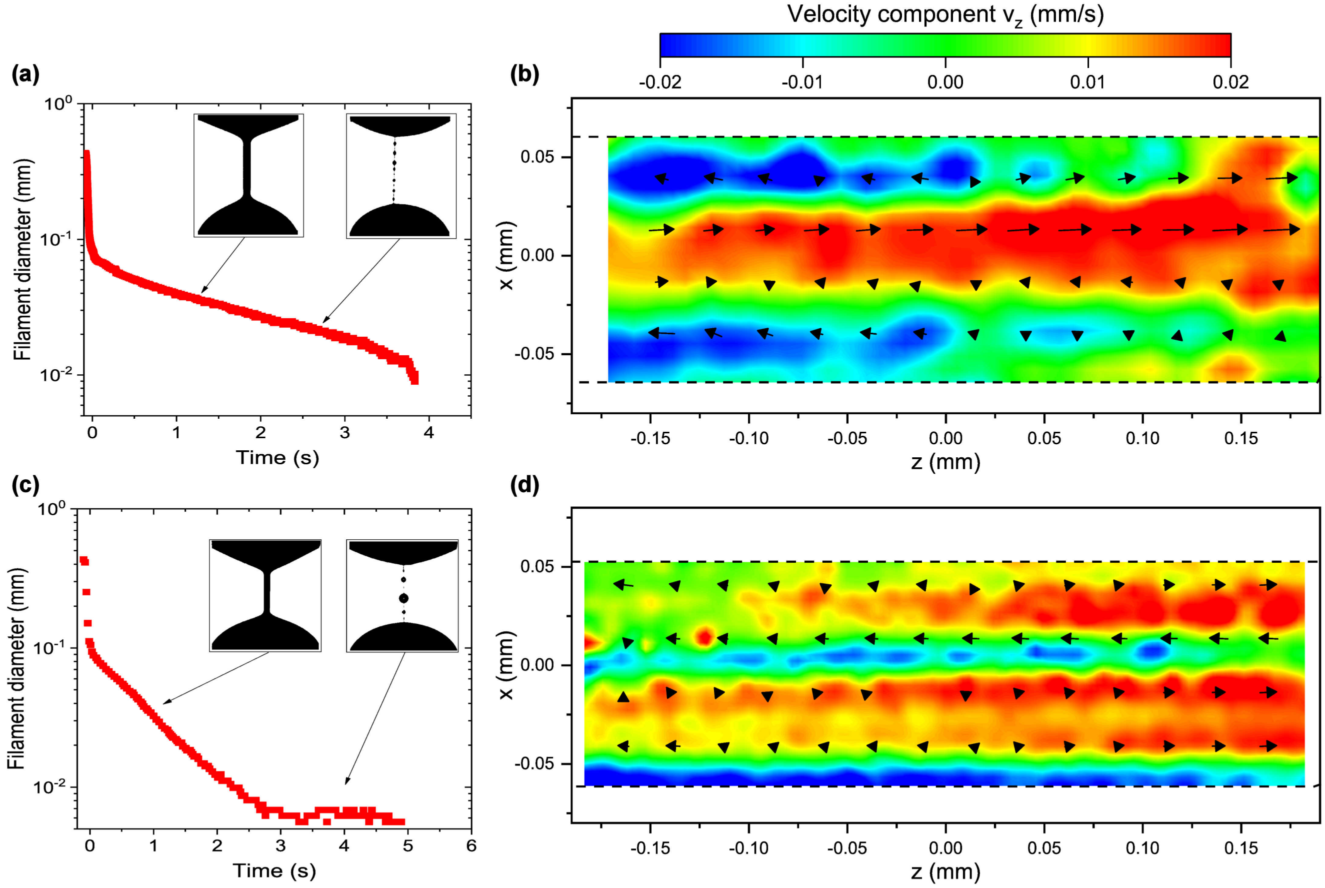

Figure S4. Heterogeneous flow inside thinning biopolymer filaments. (a) and (b) show results for an aqueous 0.14 wt% hagfish slime solution, (c) and (d) represent results for whole human saliva. (a) and (c) show diameter as a function of time as probed by CaBER, with $D_0 = 6$ mm, $h_i = 1.5$ mm, $h_f = 7$ mm, and $t_s = 40$ ms for the hagfish sample, and $D_0 = 6$ mm, $h_i = 1.5$ mm, $h_f = 6$ mm, and $t_s = 50$ ms for saliva. The inset pictures show snapshots of the cylindrical filament during thinning, the later ones displaying a beads-on-a-string structure. (b) and (d) show the velocity component in axial direction inside the filament during thinning, derived from PIV measurements. Dashed black lines represent the filament boundaries in the measured plane and arrows are to guide the eye.
